# The “Light Knife” for Gastric Cancer: Photodynamic Therapy

**DOI:** 10.3390/pharmaceutics15010101

**Published:** 2022-12-28

**Authors:** Haiyun Wang, Maswikiti Paul Ewetse, Chenhui Ma, Weigao Pu, Bo Xu, Puyi He, Yunpeng Wang, Jingyu Zhu, Hao Chen

**Affiliations:** 1 The Second Clinical College of Medicine, Lanzhou University, Lanzhou 730030, China; 2 Department of Oncology Surgery, Second Hospital of Lanzhou University, Lanzhou 730030, China; 3 Key Laboratory of Digestive System Tumor, Second Hospital of Lanzhou University, Lanzhou 730030, China

**Keywords:** photodynamic therapy, photosensitizer, gastric cancer, chemotherapy, target therapy, immunotherapy, combination therapy

## Abstract

Photodynamic therapy (PDT) has been used clinically to treat cancer for more than 40 years. Some solid tumors, including esophageal cancer, lung cancer, head and neck cancer, cholangiocarcinoma, and bladder cancer, have been approved for and managed with PDT in many countries globally. Notably, PDT for gastric cancer (GC) has been reported less and is not currently included in the clinical diagnosis and treatment guidelines. However, PDT is a potential new therapeutic modality used for the management of GC, and its outcomes and realization are more and more encouraging. PDT has a pernicious effect on tumors at the irradiation site and can play a role in rapid tumor shrinkage when GC is combined with cardiac and pyloric obstruction. Furthermore, because of its ability to activate the immune system, it still has a specific effect on systemic metastatic lesions, and the adverse reactions are mild. In this Review, we provide an overview of the current application progress of PDT for GC; systematically elaborate on its principle, mechanism, and the application of a new photosensitizer in GC; and focus on the efficacy of PDT in GC and the prospect of combined use with other therapeutic methods to provide a theoretical basis for clinical application.

## 1. Introduction

GC is one of the most common malignant tumors with an extremely thorny prognosis. According to the latest global cancer burden data (GLOBOCAN 2020), the average annual incidence of GC in the world in the past five years has been 1.806 million, including 1.397 million cases in Asia (77.4%) and 689,000 cases in China (38.2%). In addition, the incidence of GC ranks fifth, and the mortality rate is fourth [1]. Moreover, most patients (>70%) present with advanced gastric cancer (AGC) [2], with a median survival of only 12 to 15 months [3,4]. The traditional treatment methods for GC include surgery, chemotherapy, and radiotherapy. For early gastric cancer (EGC), surgical resection is the first therapeutic strategy of choice, but most GC has lost the chance of radical resection at the time of diagnosis. The efficacy of chemotherapy drugs has reached a bottleneck. The overall survival of AGC patients treated with chemotherapy alone is often less than one year, with limited efficacy and severe side effects [5,6,7]. On the other hand, targeted drugs such as anti-human epithelial growth receptor 2 (HER2) therapy can only target about 10% to 20% of the total GC population [8]. In GC patients, immune checkpoint inhibitors (ICI) provide significant survival benefits, and especially anti-PD-1 therapy can improve overall survival by 12–18 months [9]. However, anti-PD-1/PD-L1 therapy can only be effective in patients with high microsatellite instability-high (MSI-H), high PD-L1 expression, high tumor mutation burden (TMB), and Epstein–Barr virus (EBV)-positive patients. In stage IV GC patients, MSI-H/dMMR (deficient DNA mismatch repair) patients account for approximately 6% of all patients [10], and the detection rate of EBV is only about 6.2% [11]. The population with a CPS score >10 in the KEYNOTE-061 and KEYNOTE-062 studies accounts for about 25% of the overall population of AGC patients [12], so the benefited population is limited. After chemotherapy, targeted therapy, and immunotherapy, due to the emergence of drug resistance (including primary and acquired drug resistance), the curative effect is seriously affected. Currently, there is no effective treatment for this. Combined application has become a research hotspot for resisting and reversing drug resistance [13,14,15,16].

Cardiac and pyloric obstruction in GC patients is often accompanied by severe malnutrition and anemia, and the prognosis is inferior. Currently, methods such as feeding tube placement, stent placement, surgical gastrostomy, and endoscopic ultrasound-guided gastrostomy (EUS-GE) are commonly used. Nutritional tubes affect the quality of life of patients and are more often easily blocked, so they are primarily used for a short period; stent placement can quickly relieve patients’ obstructive symptoms but may lose their effectiveness because of tumor growth, food blockage, and displacement; palliative and ostomy operations are traumatic with slow recovery and poor quality life and are now usually performed in patients with longer life expectancies; EUS-GE is a new technology that has not yet been widely deployed.

*Helicobacter pylori* infection is closely associated with gastric inflammatory disease and cancer, accounting for approximately 89% of distal GC cases worldwide [17,18]. A recent large randomized controlled study suggested the important value of *H. Pylori* screening and treatment for the prevention and treatment of GC. The protective effect of *H. Pylori* therapy on GC incidence persisted 22 years after intervention (OR 0.48, 95% CI 0.32–0.71) with a fully adjusted hazard ratio of 0.62 (95% CI 0.32–0.71) after anti-*H. Pylori*, which means that only two weeks of anti-*H. Pylori* treatment can still prevent GC occurrence even after 22 years, the risk of GC incidence is significantly reduced by 52%, and the risk of GC mortality is reduced by 38% [19]. However, the continuous abuse of antibiotics has accelerated the spread of drug-resistant strains, which has brought significant challenges to eradicating *H. Pylori* using antibiotic therapy.

PDT is a technology for diagnosing and treating diseases using a photodynamic effect. It is less invasive and has a wide range of applications. It can be used not only as a local treatment method to remove tumors and relieve obstruction quickly but also as a sensitizer for chemotherapy. PDT combined with chemotherapy can solve the phenomenon of chemotherapeutic drug resistance, reduce the dose of chemotherapy drugs, and reduce toxic and side effects [20,21]. Moreover, PDT can destroy the blood vessels around the tumor and improve the efficacy of targeted drugs [22]. At the same time, it can induce the immunogenic death of tumor cells, activate the immune system, and turn “cold tumors” into “hot tumors” [23,24]. Many studies have reported that PDT can be used in combination with chemotherapy, targeting therapy, and immunotherapy to enhance efficacy [25,26,27]. In addition, PDT can eliminate *H. Pylori* [28]. Recently, with the continuous development of PDT, its application in GC has become more extensive.

## 2. New Mechanisms of PDT Anti-Tumor Therapy

PDT relies on photochemical reactions, and its three elements are the photosensitizer (PS), the light of appropriate wavelength, and oxygen dissolved in tissues [29]. The PS molecules absorb light of appropriate wavelengths and initiate photochemical reactions to destroy cells [30]. Because of the characteristics of tumor tissue and its interaction with the PS, the PS preferentially accumulates in tumor tissue and accumulates little in normal tissue, and the application of light irradiation further improves the selectivity of PDT. The ^1^O_2_ produced after PDT has a very short lifetime (about 10–320 nanoseconds), which makes its diffusion in cells only about 10 to 55 nanometers [31]. Therefore, photodynamic damage occurs where the PS is localized within the vicinity of the location [32]. PS localization is associated with cell death pathways. It has been shown that PDT can cause cell necrosis, apoptosis, or autophagy [33,34,35,36]. In general, the PS localizes to the cytoplasm to induce autophagy, to lysosomes for apoptosis and/or autophagy, and to cell membranes to cause necrosis; it induces apoptosis in the nucleus, endoplasmic reticulum, and mitochondria. It is important to note that the different forms of cell death sometimes partially overlap. Several studies have explored the mechanism by which PDT injures GC cells. For example, a study using the PS HCE6 for HCE6-mediated PDT on MKN45 cells showed that HCE6 had a significant inhibitory effect on GC cells. It was found that at a concentration of 2.62 μmol/L HCE6 and a light intensity of 3.6 J/cm^2^, MKN45 cells were almost entirely killed. RNAseq results confirmed the involvement of mitochondrial- and endoplasmic reticulum-mediated apoptosis [37]. With the deepening of research, new mechanisms of photodynamic-induced cell death are constantly being discovered. A recent study from Lanzhou University suggested that PDT induces GC cell pyroptosis through the ROS-NLRP3-CASP1-GSDMD pathway and improves the efficacy of immunotherapy for GC [38]. Shui et al. found that PDT can induce non-enzymatic lipid peroxidation in tumor cells under specific conditions, and this process does not depend on the key ALOXs or ACSL4 enzymes related to lipid metabolism in the classical ferroptosis pathway. This non-enzymatic lipid peroxidation induces tumor cells to undergo a death pathway similar to classical ferroptosis but with significant differences in iron metabolism [39]. The ways of cell death caused by PDT are shown in Table 1.

**Figure 1:**
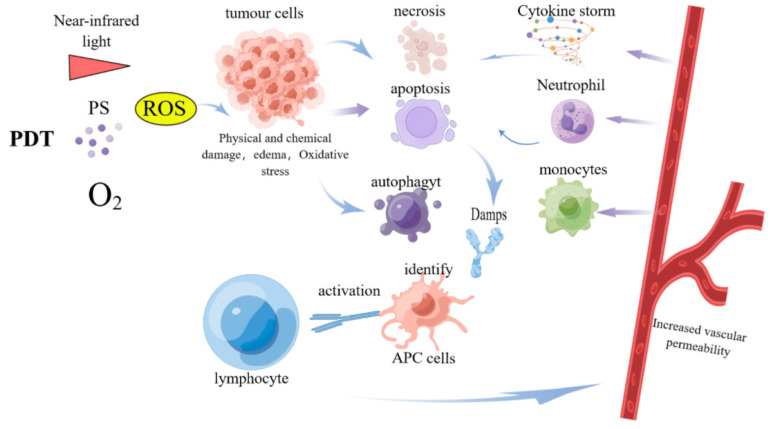
PDT-Induced Immune Effects.

PDT has been shown to induce immunogenic cell death (ICD) [40,41], which can release damage-associated molecular patterns (DAMPs). DAMPs are, essentially, molecules that remain within cells under normal conditions but are exposed on the cell surface or released from cells following physical, chemical, or biological damage. After DAMPs are exposed or released, they interact with various pattern-recognition receptors (PRRs), such as RIG-I-like receptors, NOD-like receptors, and TOLL-like receptors [42]. DAMPs usually have strong immunostimulatory properties, causing the maturation and activation of antigen-presenting cells (APCs) to present and process antigens [43]. Central to this process is the presentation of antigenic peptides by dendritic cells (DCs) to CD8^+^ T cells of the adaptive immune system via major histocompatibility complex class I molecules (MHC I) [44]. It has been shown that PDT-induced ICD can induce the exposure or release of DAMPs, such as HSP70, HMGB1, and ATP [45,46,47], and then activate adaptive immune responses, thus effectively controlling systemic tumors and forming long-term anticancer immunity [48]. However, because of the limitations of PDT itself and the poor immunogenicity and immunosuppressive microenvironment of tumor tissue, it is difficult for PDT to obtain strong and durable adaptive antitumor efficacy in cancer immunotherapy. Some researchers have searched for the optimal PDT conditions to induce systemic immunity and have developed endoplasmic reticulum-targeted PDT, which induced intense endoplasmic reticulum stress and calreticulin (CRT) exposure on the cell surface and stimulated dendritic cells’ antigen-presenting function, enhancing immunogenic cell death [49]. These studies provide a firm theoretical basis for PDT combined with immunotherapy. The immune effect induced by PDT is shown in Figure 1.

After the PS enters the tumor tissue and is irradiated with laser light of a specific wavelength, a photochemical reaction is initiated to generate a large amount of ROS. ROS can directly kill tumor cells, and the damaged tumor cells induce the release of various cytokines and chemokines and then recruit inflammatory cells related to innate immunity to generate local inflammatory responses. Moreover, PDT can induce ICD in tumor cells and expose or release DAMPs. DAMPs stimulate the maturation and activation of APCs. Then, APCs phagocytize and present tumor-specific antigens to T lymphocytes, and these activated T cells enter the systemic circulation to mediate a broader systemic anti-tumor immune effect.

## 3. Application of New PSs in GC

### 3.1. Comparison of PS in the Treatment of GC

The PS with the most extensive history of clinical application in cancer treatment is the hematoporphyrin derivative (HPD). The purified form of HPD is sodium porphyrin, later known as Photofrin. The HPD dosage for GC is 2.5–5 mg/kg, that of Photofrin is 1.3–2.5 mg/kg, and the therapeutic light wavelength is 630 nm. Although sodium porphyrin is still the most widely used PS, it has severe skin photosensitivity and low absorbance at 630 nm, resulting in insufficient tissue penetration depth. 5-Aminolevulinic acid (5-ALA) is classified as a second-generation PS with a higher chemical purity and singlet oxygen generation rate. Its maximum absorption wavelength range is 650–800 nm, and the wavelength is longer. The depth of action is thus increased. In addition, its photosensitive period is short, and the side effects are fewer. There is a long time interval between administration and irradiation, so the PS has enough time to diffuse from normal tissues. Usually, the PS is intravenously dripped 48 to 72 h before irradiation. In treating GC, PDT is mainly carried out using a gastroscope. Under the guidance of the gastroscope, the optical fiber is positioned on the lesion for irradiation. Usually, the energy density of laser irradiation is 200–300 J/cm^2^, and the output power of optical fiber is 800–1000 mw. According to the scope of the lesion, it is divided into 2–3 irradiations, and each irradiation time is 10–20 min.

Different PSs have different physical and chemical properties, activation wavelengths, and patterns of photosensitization. For example, one study [50] used di-sulphonated aluminum phthalocyanine (AlS2Pc) and 5-ALA as PSs to study the photosensitization pattern in a normal rat stomach. AlS2Pc causes full-thickness photosensitization of the gastric wall. After exposure to light, full-thickness damage occurs, while the 5-ALA photodynamic effect is confined to the mucosa. Therefore, in PDT for patients with GC, issues such as PS characteristics, wavelength, light intensity, and depth of killing need to be considered to avoid complications such as gastric perforation and gastric bleeding. In vitro, in vivo, and clinical studies of various kinds of PS-mediated PDT for GC reported in the literature are shown in Table 2, Table 3 and Table 4 [51,52,53].

### 3.2. The Effect of Using New PSs in PDT for GC

Several hypotheses have been proposed to explain the selectivity of PSs for tumor tissue, such as enhanced permeability and retention effects. This hypothesis means that because of the formation of a large number of new blood vessels in the local area of the tumor, the endothelium of these blood vessels is not perfect, resulting in leakage. Furthermore, the local lack of perfect lymphatic drainage leads to the accumulation of PSs [62]. It is also theorized that the porphyrin level in tumor-associated macrophages is nine times higher than in tumor cells, and macrophages absorb and degrade low-density lipoprotein (LDL). The interaction of PSs with LDL is an essential factor leading to the localization of PSs in tumor tissues [63]. One of the critical directions for developing new PSs is to further improve the selective aggregation of PSs in tumor tissues. With this in mind, researchers have put forward ideas such as targeting PSs, which relies on targeting carriers to make PSs adhere to the tumor cells or receptors expressed by specific tumors. The carriers include nanoparticles, liposomes, micelles, carbon nanotubes, or specific ligands such as monoclonal antibodies, polypeptides, low-density lipoproteins, and various carbohydrates. For example, some studies combine PSs with liposomes to determine whether PS liposomes can improve the therapeutic effects of PDT. Igarashi and others encapsulated PSs in multilamellar liposomes. They showed, using a mouse model of human GC xenografts, that compared with simple photosensitizers (PF), the photosensitizer liposome (liposomal Photofrin, LPF) levels of the PSs in tumor tissue were 2.4 times higher than those of the PF group and nearly equal to those in the skin. The area of tumor necrosis in the LPF group (69.6%) was significantly increased compared with the PF group (39.6%) after irradiation [57]. This study suggests that the liposomization of PSs increases the specific aggregation of PSs in tumor tissues and enhances the therapeutic effect of PDT. Namiki et al. integrated the lipophilic PS CE6-Na into the lipid bilayer to develop a novel photosensitizing liposome (PSSL). It also demonstrated that PSSL has a more substantial photodynamic effect in GC cell lines and mouse models [54].

The development of drug delivery vehicles further aids PDT. Nanoparticle-based drug delivery systems have shown better availability and fewer toxic side effects than pure drugs. In a novel study, the researchers used the cell membranes (CM) of SGC7901 cells to modify silica nanoparticles (SLN) to construct a drug-loading platform CM/SLN that can specifically target and bind to SGC7901 cells and finally loaded with the PS chloride e6 (CE6). Cell experiments showed that CM/SLN/CE6 had good dispersion and stability and could target junction and SGC7901 cells. Further in vivo experiments showed that CM/SLN/CE6-mediated PDT was more effective than SLN/CE6 [64].

In addition, the natural cell membrane provided by exosomes enhances the biocompatibility and transcellular permeability of nanocarriers. In one study, high-purity exosomes were obtained from GC patients using a non-invasive method, and then exosomes were integrated with PSs to prepare Exo-PMA/Au-BSA@Ce6, which can enter cancer cells efficiently and, due to its membrane structure and antigenic action, delays the endocytosis of macrophages, thus prolonging the blood circulation time. In vivo experiments in MGC-803 tumor-bearing, nude mice show that Exo-PMA/Au-BSA@Ce6 nanocarriers target tumor cells with deep penetration and better retention in tumors [65].

## 4. Diagnostic Role of Photodynamics in GC

The principle of photodynamic diagnosis (PDD) is to use the targeted aggregation of PSs at the tumor site and then use a specific wavelength of light to excite the PSs in the tumor to generate a fluorescence effect, thereby facilitating a specific diagnosis of tumor tissue. It can be used to judge the margin of tumor resection and lymph node metastasis in patients with GC during surgery, determine the degree of lymph node dissection, and formulate individualized treatment strategies. 5-ALA, a precursor of PS currently used for PDD, is a naturally occurring amino acid derivative that acts as a precursor of protoporphyrin IX (PpIX). When excited under irradiation with specific wavelengths (mainly visible blue light with a wavelength of 375–475 nm), it emits red fluorescence, while benign lesions do not. This property of PpIX can be exploited to precisely identify cancer cells [66,67]. In the study of Namikawa et al., intraoperative PDD was performed on 26 lesions in 21 GC patients. Red fluorescence-positive lesions were compared with the pathological findings. The sensitivity, specificity, and accuracy of ALA-PDD were 57.7%, 100%, and 66.7% [68], respectively, confirming the role of ALA-PDD in diagnosing GC.

**Figure 2:**
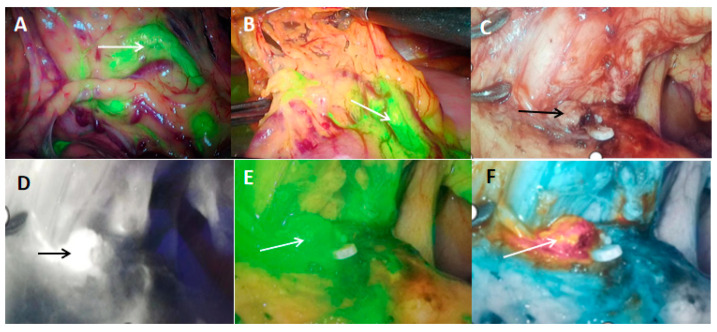
Indocyanine green (ICG) near-infrared imaging for tumor and lymph node localization in laparoscopic radical gastrectomy. (**A**,**B**) arrow refers to the metastatic lymph nodes; (**C**–**F**) are ICG near-infrared light imaging of the lymph nodes behind the pancreas head, showing conventional cold light imaging, black and white mode, green mode, and color fluorescence mode, respectively.

Indocyanine green (ICG) is a near-infrared light contrast agent with good biocompatibility. It can be excited by light with a wavelength of 750–800 nm and emit near-infrared light with a longer wavelength to achieve tissue and organ imaging and intraoperative localization of tumor tissue and metastatic lymph nodes, as shown in Figure 2. One study used ICG-derivative nanoparticles (ICGm) as PSs. Animal experiments showed that ICGm selectively accumulated in tumor tissues, and peritoneal tumors in mice could be observed through the abdominal wall. Disseminated nodules were reduced after PDT. ICGm are of great value in the diagnosis and treatment of the peritoneal dissemination of GC [69].

## 5. Effects of PDT on GC

### 5.1. PDT for Early Gastric Cancer (EGC)

The traditional treatment method for EGC without the risk of lymph node metastasis is endoscopic resection (ER). In the early 1990s, Japan approved PDT for treating EGC, including patients who could not undergo conventional ER therapy because of submucosal infiltration or ulcer scarring. Later, the clinical trials of PDT for EGC showed excellent curative effects. In a multicenter study on PDT for early-stage cancer in Japan in 1990, including 120 EGC cases, 77.3% of EGC patients showed complete remission (CR) after PDT, which indicated that PDT has the potential to cure early cancer lesions [59]. Kato et al. used PDT to treat 58 cases of superficial cancer, and 48 cases achieved (82.8%) complete remission [60]. Mimura et al. applied PDT to 27 patients with EGC. The results showed that 88% of the 24 evaluable patients achieved complete remission (CR), and the effective rate was 100%. CR was seen in all non-ulcerated superficial depressed lesions and lesions with a tumor diameter of less than 2 cm, with minimal adverse effects [61].

With the improvement of the light source and the advancement of technology, PDT has been proven to be a safe and effective method for treating EGC, not only for the intramucosal type but also for the submucosal infiltrating type. In 2016, Oinuma et al. conducted a retrospective study on the use of PDT for GC in 18 centers in Japan. Among 57 patients with EGC with a tumor ≤ 2 cm and ulcer scars that were not unsuitable for surgery, 42 (73.7%) patients achieved complete remission [53]. Liu reported that 7 cases of early esophageal cancer and GC were treated with Photofrin PDT and HPD-PDT, and all cases were cancer-free during 15 years of follow-up [70].

There are also some rare EGCs where a routine gastric biopsy is diagnosed as GC without any visible lesions. However, a repeat biopsy is negative, and gastrectomy is usually recommended for patients with invisible GC. In a prospective study of 22 patients with invisible GC over a 10-year period who underwent PDT with ALA in the area of the biopsy, at a 6-year follow-up, three patients died from causes unrelated to GC. Four patients developed mucosal cancer, and the remaining fifteen patients had no evidence of GC recurrence, lymph node involvement, or metastasis during follow-up [52].

### 5.2. PDT for the Treatment of Locally Advanced and Peritoneal Metastatic GC

There have been many reports on diagnosing and treating advanced gastric cancer (AGC) using PDT. For example, a study on the correlation between the clinical efficacy of PDT for AGC and the histopathological typing and grading of GC conducted by Yu et al. included 129 patients with stage III-IV AGC. After 2 to 3 courses of PDT treatment, there were 3 cases of complete remission (CR), 54 cases of significant remission (SR), 67 cases of minor remission (MR), and 5 cases of no remission (NR). The total effective rate was 96% and showed that the treatment effect was not related to the histopathological type or grade of GC [71]. Song and others performed PDT on 140 patients with AGC, and the results showed that the total effective rate was 89.3% (*p* < 0.005) in the short term, and after four years of follow-up, 6 cases (4.3%) survived [72]. Therefore, he believes that the short-term efficacy of PDT in the treatment of AGC is suitable, but the long-term efficacy is not optimistic.

When AGC is complicated with peritoneal metastasis, the existing treatment methods are ineffective, and the prognosis is poor. PDT may be a potential treatment for the peritoneal dissemination of GC. Kishi et al. conducted in vivo studies on a nude mouse peritoneal metastasis model of a human GC MKN-45 EGFP cell line using different doses of talaporfin (2 J/cm^2^, 5 J/cm^2^, and 10 J/cm^2^)-mediated PDT. They found that after intraperitoneal injection of talaporfin, the accumulation intensity of peritoneal metastatic nodules is higher than that of normal tissues. The pathological response rates after treatment range from 20.8 to 64.4. Therefore, Kishi believes that talaporfin-mediated PDT may be an effective treatment for patients with advanced gastric adenocarcinoma and metastatic peritoneal nodules [58]. Some researchers have also pointed out that the combined use of PDT, surgery, chemotherapy, and radiotherapy is likely to improve the effectiveness of treating peritoneal cancer [73].

**Figure 3:**
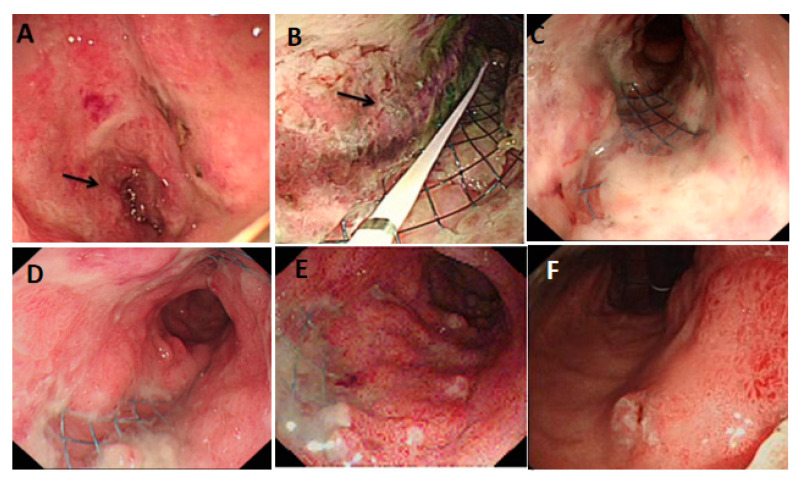
A patient with AGC with pyloric obstruction received PDT. (**A**) Gastroscope shows tumor invasion from the lesser curvature to the pylorus, and the arrow indicates the pyloric stenosis; (**B**) PDT, the arrow points to the visible optical fiber inserted into the original stenosis (metal stent area), and the mucosa is white and necrotic after irradiation; (**C**) after one week of PDT, the local mucosa was gray and white, showing ischemic changes; (**D**) after one month of PDT, the lumen at the pylorus was unobstructed; (**E**) the tumor tissue from the anterior wall of the lesser curvature to the pylorus was smaller than before; (**F**) the lumen was still unobstructed after five months of PDT.

**Figure 4:**
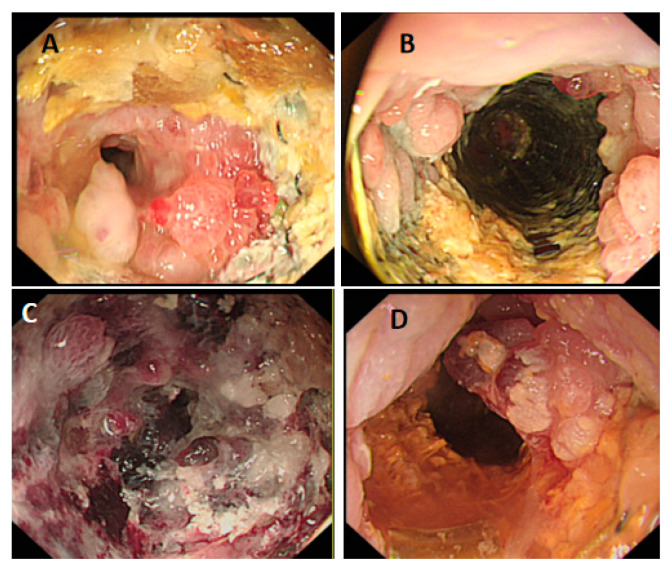
A patient with AGC receiving PDT. (**A**) Before treatment, atresia of the cardia can be seen; (**B**) it can be seen that the lumen is unobstructed after stent placement combined with PDT; (**C**) after one week of treatment, much gray-white necrotic tissue can be seen; (**D**) after three months of treatment, necrotic tissue can be seen to fall off, the lumen is unobstructed.

Therefore, PDT is an essential option for patients with AGC who have lost the opportunity for surgery or are refractory to chemotherapy. The application of PDT in patients with AGC complicated with cardiac or pyloric obstruction has not been reported yet. The Cancer Center of the Second Hospital of Lanzhou University used metal stents combined with PDT to quickly relieve the symptoms of pyloric and cardiac obstruction in patients. The patient can eat normally, recover quickly, and control tumor progression effectively. The process of treatment is shown in Figure 3 and Figure 4.

## 6. Combined Treatment of PDT

### 6.1. Combined Use of PDT and Chemotherapy Drugs

Chemotherapy drugs are non-specific cytotoxic drugs. While killing cancer cells, they are also toxic to normal cells throughout the body and are prone to drug resistance, which limits their clinical application. Some studies have found that PDT combined with chemotherapy can produce synergistic anti-tumor effects, improve efficacy, reduce the therapeutic dose of chemotherapy drugs, and reduce toxic side effects. For example, a study compared the synergistic anticancer activity of AlPcS4 (a photosensitizer)-mediated PDT combined with different low-dose chemotherapeutic drugs on SGC-7901 GC cells. The research results showed that photodynamics can increase the intracellular uptake ability of AlPcS4, improve the apoptosis-inducing ability, and prolong the apoptosis-inducing time. Moreover, a low dose of chemotherapeutic drugs may have lower toxic and side effects and has an apparent synergistic effect [74].

Long-term chemotherapeutic drugs can induce multidrug resistance (MDR) [75], which has become an important factor limiting the efficacy and application of some chemotherapeutic drugs. Drug efflux by transmembrane P-glycoprotein (Pgp) is one of the leading causes of cellular MDR [76]. The principle of PDT therapy is different from chemotherapy, and it may be a potential method for the treatment of tumor MDR. A study reported the therapeutic effect of a novel porphyrin-based PS DTP on human paclitaxel-resistant human GC cell line MGC803/PA. The results showed that Pgp overexpressed in the MDR cell line could not expel DTP from the cells, and DTP-mediated PDT could eradicate GC cells regardless of whether they expressed MDR or not. Moreover, the proliferation ability of MGC80/PA cell lines induced by TDP-PDT was significantly impaired [55].

Preclinical studies have shown the advantages of PDT combined with chemotherapy in treating GC. At present, it has been used in clinical work. A 67-year-old patient with AGC developed tumor progression after chemotherapy, with pyloric obstruction and biliary obstruction, eventually leading to nutritional and functional disorders. The patient was treated with duodenal and biliary metal stents combined with PDT, quickly relieving obstructive symptoms. Symptoms such as nausea, vomiting, abdominal pain, abdominal distension, and jaundice were relieved, and a regular liquid diet was started on the second day after treatment. The quality of life was significantly improved [77].

### 6.2. Combined Use of PDT and Targeted Drugs

Specific antibody therapy against tumor cells is combined with a PS to form a new type of antibody–photoabsorber conjugate (APC). After APC is intravenously injected into the body, near-infrared light is irradiated to the lesion site, and the conjugate undergoes a chemical reaction to destroy the tumor cells. At the same time, irradiation in regions without the APC will not cause damage. It has the characteristics of both targeted drugs and PSs. A targeted PDT modality has a more substantial selectivity and killing effect on tumor tissues, does not affect normal tissues, and has fewer side effects. It is called photoimmunotherapy (PIT) or near-infrared photoimmunotherapy (NIR-PIT).

HER2 is overexpressed in many GC patients. The anti-HER2 antibody trastuzumab is an effective supplement to standard chemotherapy in HER2-positive GC patients. One study developed a new photosensitizer-targeted drug conjugate (Trast-Porph) by combining porphyrin with trastuzumab for targeted PDT in HER2-positive GC. In vitro data showed that Trast-Porph specifically binds to HER2-positive cells, co-localizes with the lysosomal marker LAMP1, and accumulates in cells. Its lethality to HER2-positive GC cells is twice that of trastuzumab alone. A nude mouse xenograft model established using a HER2-positive GC cell line also showed that Trast-Porph-based photodynamic-antibody therapy was more cytotoxic than trastuzumab and delayed tumor growth, and 14 days later, the tumor volume was reduced by about 17% [56]. A novel APC was also prepared by coupling a human monoclonal antibody (Ab) against carcinoembryonic antigen (CEA) to a near-infrared light-excitable phthalocyanine derivative IRDye700DX. The study found that CEA-positive cells CHO-CEA were almost wholly killed after light exposure. In contrast, CEA-negative cells were utterly unaffected, indicating that their phototoxicity depends on the expression of CEA on target cells. Further animal experiments showed that the tumor size was about twice as small as that of the antibody-only group. Therefore, this novel conjugate has high specificity and potent cytotoxicity for CEA-positive tumor cells [78]. In addition to APC, PDT combined with target therapy for VEGFR2 was applied in clinic. A 72-year-old patient with AGC who received PDT in combination with apatinib was reported in the literature. At the 7-month follow-up, tumor marker levels were reduced, and there was no recurrence or metastasis [79].

There are also studies based on this concept that have developed an *H. Pylori*-targeting PDT system p3SLP, which is composed of multiple 3’-sialyllactose (3SL)-conjugated polylysine (PLL) and photosensitizer (PheoA) compositions. p3SLP forms a complex targeting *H. Pylori* through specific interactions with *H. Pylori* membrane adhesin (SabA) and destroys biomolecules such as DNA and cell membranes through ROS generated under laser irradiation. In *H. Pylori*-infected mice, p3SLP had a similar antibacterial therapeutic effect to the triple antibiotic regimen without adverse side effects on normal tissues and intestinal flora. Moreover, the bactericidal mechanism of PDT and antibiotics is different, and the possibility of causing drug resistance is slight. PDT provides a new idea for the treatment of *H. Pylori* [28].

### 6.3. PDT Combined with Immunotherapy

PDT can elicit various effects in the tumor microenvironment, leading to the infiltration of various immune cells into the treatment site [80]. PDT has also been implicated in activating different immune phenomena, such as the acute phase response, the complement cascade, and cytokine/chemokine production [81,82]. Moreover, PDT can activate “anti-tumor adaptive immunity.” Some researchers have used PDT to develop tumor vaccines or have combined it with other immunotherapies to enhance anti-tumor effects, such as using tumor cell lysates produced with in vitro PDT to develop tumor vaccines. Multiple studies have shown that immunization with PDT-killed tumor cells, such as EMT6, P815, SCC VII, and H22, induces robust anti-tumor immune responses [83,84]. However, both UV- and PDT-generated tumor cell lysates can induce DC cell maturity, but only PDT-generated lysates were able to activate DCs to express IL-12, which was superior to the tumor cell lysates generated using UV and ionizing irradiation or freeze-thawing [85].

The effectiveness of PDT in enhancing anti-tumor immunity has begun to be studied in phase I clinical trials in cancer patients [86]. Abdel-Hady et al. showed that patients with vulvar intraepithelial neoplasia (VIN) unresponsive to ALA-PDT were more likely to develop tumors than those who responded to ALA-PDT. Responders had more CD8^+^ T cell infiltration in tumor tissue than non-responders. This research suggests long-term antitumor immune effects after PDT [87]. 

As mentioned earlier, PDT can elicit an anti-tumor immune response, but its immune response may not be strong enough to eliminate tumors. The immune adjuvant is referred to as adjuvant, a non-specific immunoproliferative agent. It can enhance the body’s immune response to antigens or change the type of immune response after entering the body [88,89]. A study simultaneously loaded the PS Ce6 and the toll-like receptor-7 agonist imiquimod, an immune adjuvant, on nanoparticles (UCNPs) under near-infrared (NIR) irradiation, which promoted strong anti-tumor immune responses [90]. A study by Zheng et al. also showed that glycosylated chitosan (a new immune adjuvant) combined with PDT is more effective than PDT alone [91,92].

A clinical study used immunotherapy combined with PDT for AGC. Two elderly patients with AGC (89 and 92 years old) had no response to initial PDT alone. After the patients received injections of more than 10^9^ activated T lymphocyte-based autologous immune cells, PDT was performed again. As a result, the patients’ bleeding symptoms were significantly improved, the 89-year-old patient survived for 14 months and the 92-year-old patient survived for more than 32 months, and the survival period was significantly longer than expected [93]. Currently, there are no relevant animal experiments or clinical studies on PDT combined with ICI for treating GC.

### 6.4. Combined Use of PDT and Hyperthermia

It has been reported in the literature that high levels of ROS were generated in RGK36 and RGK45 cells at a high temperature of 42 °C for 1 h, which further modulated the expression of heme carrier protein-1 (HCP-1) and ABCG2 to enhance photodynamic phototoxicity. Furthermore, hyperthermia produces mitochondrial reactive oxygen species (mitROS), which up-regulate the uptake transporter HCP-1 of porphyrins and increase the tumor-specific accumulation of porphyrins, thereby enhancing the killing effect of PDT on tumors [94]. Animal experiments and clinical trials on PDT and hyperthermia have not yet been carried out.

## 7. Conclusions and Outlook

PDT can be used to treat superficial diseases such as precancerous lesions, carcinoma in situ, or superficial tumors. It has shown sound therapeutic effects in many clinical treatments for EGC and can be used as an effective supplementary treatment for endoscopic treatment. For patients with inoperable AGC, especially those with peritoneal metastasis and cardiac or pyloric obstruction, PDT can be used for palliative treatment to improve the patient’s quality of life. The fluorescent effect produced by some PSs can identify tumor tissue and metastatic lymph nodes during operation, making the operation easier. Moreover, PDT induces ICD, activates the adaptive immune system, exerts long-term anti-tumor effects similar to “cancer vaccines”, and plays a synergistic therapeutic role in combination therapy with immunotherapy. Combined with targeted drugs, especially NIR-PIT, it is a promising new anticancer therapy, known as the fifth cancer treatment. Some new PSs still have a deadly effect on chemotherapy-resistant GC cells, and combined use reduces the dose of chemotherapy and improves the therapeutic effect. In addition, PDT can be used repeatedly and can be used before or after chemotherapy and surgery with mild side effects. It is worth noting that the current research on PDT, such as the development of new PSs or new drug-loading platforms, and its combined use with other therapeutic methods is still mainly in the primary research stage, and there is a lack of large-scale clinical randomized controlled trials to prove its clinical efficacy. With further research on the molecular mechanism of PDT, we expect further progress in the synergistic treatment of PDT with chemotherapy, targeting therapy, and immunotherapy, which will provide a safe and effective new method for tumor treatment.

## Figures and Tables

**Table 1:** PDT-induced cell death modality and mechanisms.

Mechanism	Mode of Action	References
Necrosis	Substantial membrane photodamage	Ref. [33]
Apoptosis	Mediated Bcl-2 loss and promoted the release of Ca (2+) from the endoplasmic reticulum	Ref. [34]
Autophagy	Targets the mTOR signaling network	Refs. [35,36]
Pyroptosis	Photodynamics induced pyroptosis through ROS-NLRP3-CASP1-GSDMD pathway	Ref. [38]
Ferroptosis	Non-enzymatic lipid peroxidation	Ref. [39]

**Table 2:** In vitro study of PDT for GC.

Author	Photosensitizer	Year	Activation Wavelength	Cell Line	Efficacy
Tan YK [37]	HCE6	2019	660 nm	MKN45	Completely kill the cell
Namiki Y [54]	PSSL	2003	300–750 nm	MKN-45, MKN-74 NUGC4, HSC-43	The cell killing rate of PSSL was 53 times higher than that of CE6-NA photosensitizer
Chen J [55]	DTP	2015	650 nm	MGC803 MGC803/PA	Eradicate gastric cancer cells whether or not they express MDR
Wang X [51]	5-ALA	2016	375–400 nm	MKN-45	Significantly inhibit proliferation and promote apoptosis
Korsak B [56]	Trast:Porph	2017	-	Her2-Positive cells	Higher selectivity and cytotoxicity than trastuzumab

**Table 3:** In vivo study of PDT for GC.

Author	Photosensitizer	Year	Activation Wavelength	Cell Line	Modeling Animal	Efficacy
Igarashi A [57]	^1^ LPF	2003	630 nm	MT-2	BALB/c nude mice	Enhanced selective aggregation of photosensitizers in tumor tissue
Kishi K [58]	Talaporfin	2010	664 nm	MKN-45	BALB/c nude mice	Possibly effective in patients with advanced GC and metastatic peritoneal nodules
Namiki Y [54]	PSSL	2003	300–750 nm	MKN-45 MKN-74 NUGC4 HSC-43	BALB/c nude mice	Stronger photodynamic effect than CE6-NA

^1^ Liposomal Photofrin.

**Table 4:** Clinical study of PDT for GC.

Author	Photosensitizer	Year	Activation Wavelength	Tumor Types	Number of Patients	Efficacy
Kato H [59]	Photofrin II(DHE)	1833	630 nm	Early GC	120	CR77%
Kato H [60]	Photofrin	1993	630 nm	Superficial cancer	58	CR83%
Mimura S [61]	Photofrin II	1996	630 nm	Early GC	27	CR88%
Rabenstein [52]	ALA	2008	635 nm	Early GC	22	CR68%
Oinuma T [53]	Photofrin, Talaporfin, ALA, HPD	2016		Early GC	57	CR74%

## Data Availability

Not applicable.
